# Overview of Single-Molecule Speckle (SiMS) Microscopy and Its Electroporation-Based Version with Efficient Labeling and Improved Spatiotemporal Resolution

**DOI:** 10.3390/s17071585

**Published:** 2017-07-06

**Authors:** Sawako Yamashiro, Naoki Watanabe

**Affiliations:** 1 Laboratory of Single-Molecule Cell Biology, Kyoto University Graduate School of Biostudies, Kyoto 606-8501, Japan; watanabe.naoki.4v@kyoto-u.ac.jp; 2 Department of Pharmacology, Kyoto University Graduate School of Medicine, Kyoto 606-8501, Japan

**Keywords:** single-molecule imaging, actin dynamics, live cell imaging

## Abstract

Live-cell single-molecule imaging was introduced more than a decade ago, and has provided critical information on remodeling of the actin cytoskeleton, the motion of plasma membrane proteins, and dynamics of molecular motor proteins. Actin remodeling has been the best target for this approach because actin and its associated proteins stop diffusing when assembled, allowing visualization of single-molecules of fluorescently-labeled proteins in a state specific manner. The approach based on this simple principle is called Single-Molecule Speckle (SiMS) microscopy. For instance, spatiotemporal regulation of actin polymerization and lifetime distribution of actin filaments can be monitored directly by tracking actin SiMS. In combination with fluorescently labeled probes of various actin regulators, SiMS microscopy has contributed to clarifying the processes underlying recycling, motion and remodeling of the live-cell actin network. Recently, we introduced an electroporation-based method called eSiMS microscopy, with high efficiency, easiness and improved spatiotemporal precision. In this review, we describe the application of live-cell single-molecule imaging to cellular actin dynamics and discuss the advantages of eSiMS microscopy over previous SiMS microscopy.

## 1. Introduction

Remodeling of the cortical cytoskeleton plays a central role in cell locomotion, cytokinesis, endocytosis, phagocytosis and tissue organization. To elucidate the regulation of cell morphogenesis, real-time monitoring of molecular behavior in the actin cytoskeleton offers tremendous potential. The development of live-cell fluorescence single-molecule imaging has opened a window for the direct viewing of assembly and remodeling processes of the actin filament network. Many of these processes have turned out to occur much faster than predicted by in vitro biochemical data [1]. Live-cell single-molecule imaging has provided critical information on not only remodeling of the actin cytoskeleton [1,2,3], but also the motion of plasma membrane proteins [4,5] and dynamics of molecular motor proteins [6,7].

Prior to the development of this approach, two methods were mainly employed to monitor the dynamics of actin filament turnover in live cells. In one method, researchers observed the site of incorporation of microinjected labeled actin probes [8,9]. These studies revealed that actin incorporates fastest into the tip of pseudopods, such as filopodia and lamellipodia. The other methods are photoactivation of fluorescence (PAF) of caged fluorescent actin and fluorescence recovery after photobleaching (FRAP) of fluorescently-labeled actin. In an early FRAP study [10], the retrograde actin flow, which is the centripetal movement of the actin network ubiquitously observed at the periphery of cultured cells, was visualized. Another study employing photoactivation of caged fluorescent actin [11] further revealed that the majority of F-actin disassembles before reaching the lamellipodium base in migrating fish keratocytes. Although these seminal studies revealed the dynamic actin filament turnover and the retrograde actin flow in lamellipodia, there was a long-standing debate as to whether treadmilling governs [12,13,14] actin filament turnover in lamellipodia or actin undergoes frequent disassembly and reassembly within the body of lamellipodia [11]. A further development of FRAP and PAF is fluorescence localization after photobleaching (FLAP), in which actin are labeled with two distinct fluorophores: one to be photobleached and the other to act as a reference label [15]. The use of a reference fluorophore allows the tracking of the distribution of the labeled actin by simple image differencing and thus enables measurement of fast redistribution dynamics [15,16]. As discussed in detail in previous studies [1,17], FRAP, PAF and FLAP experiments may exhibit potential problems, such as low spatial resolution, insufficiency in monitoring subpopulation, local reincorporation of disassembled probes and photodamage during the photobleaching procedure. These problems may have given rise to the distinct results and interpretations in the FRAP and PAF studies. 

On the other hand, single-molecule imaging directly visualizes assembly, dwell time and movement of individual fluorescently labeled molecules. One can also follow the change in the molecular behavior over time by only intermittently acquiring the images. In 1998, fluorescent speckle microscopy (FSM), which visualizes movement of cytoskeletal polymers in living cells with higher resolution than the above microscopy techniques, was introduced [18]. The principle of FSM is that incorporation of fluorescently labeled subunits at low concentrations provides fiduciary marks, which are referred to as speckles, on polymers. By further lowering the density of the labeled actin probes, we were able to demonstrate the actin “speckles” consisting of a single enhanced green fluorescent protein (EGFP) tagged actin molecule in live cells [19]. We call our observation target “single-molecule speckle (SiMS)” to distinguish from the “speckles” characterized by Waterman-Storer and her colleagues [18,20]. In the original FSM method, “speckle” was claimed to contain 2 to 10 fluorescent molecules in resolvable image units whereas we track the behavior of individual fluorescent molecules. Through statistic analysis of a large number of molecular events, researchers can extract exact kinetic parameters in molecular assembly and disassembly reactions in vivo. To correctly perform SiMS analysis in live cells, one must carefully design the observation conditions to minimize the photodamage of the sample due to the strong illumination required for single-molecule visualization and error tracking of SiMS behavior. A decade of experience has steadily improved the feasibility of SiMS microscopy [21,22]. Especially the use of electroporation of fluorescent proteins for cell labeling and the introduction of new photostable chemical dyes extends the applications of SiMS microscopy to cellular structure that could not previously be imaged using this technique.

In this article, we briefly review principles and applications of SiMS microscopy. We also summarize our recent progress in SiMS methods to provide background information for our accompanying paper introducing new near infrared actin probes.

## 2. Principles of Single-Molecule Speckle (SiMS) Microscopy

**Figure 1:**
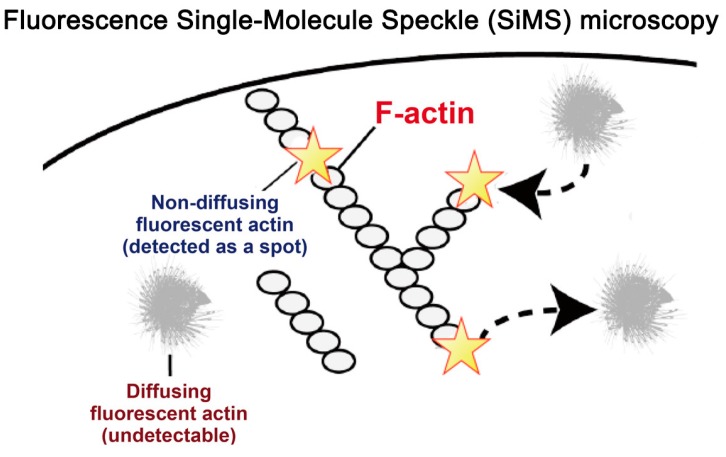
A schematic diagram of the principles of Single-Molecule Speckle (SiMS) microscopy.When the expression level of EGFP-actin is sufficiently low, signals from EGFP-actin assembled with F-actin add up in a small spot on the CCD with long exposure time. In contrast, signals from a freely diffusing EGFP-actin in the monomeric form are blurred on the image.

SiMS microscopy is an approach to monitor complex behaviors of proteins in living cells with considerably high resolution. In the original SiMS analysis, which was first introduced in 2002 [19], individual EGFP-actin molecules were visualized in cells expressing a very low level of EGFP-actin under the control of a defective cytomegalovirus (CMV) promoter. EGFP-actin either co-assembles with filamentous actin (F-actin) or exists as monomeric actin (G-actin) in cells. When the expression level of EGFP-actin is sufficiently low, signals from EGFP-actin assembled with F-actin add up in a small spot on the charged-coupled device (CCD) with long exposure time up to 2 s. In contrast, signals from a freely diffusing EGFP-actin in the monomeric form are blurred on the image. The diffusion constant of actin monomer is 3–6 μm^2^·s^−1^ [23]. Monomeric probes travel through a path of a few micro meters during the 2-s exposure time. Therefore, only EGFP-actin in the F-actin state can be visualized as single-molecules (Figure 1). With this simple principle, cytoskeletal proteins and their associating proteins are visualized as single-molecules in a state specific manner [2,3,19,24,25,26,27,28]. The SiMS method also enables tracking of the membrane-bound proteins along the plasma membrane [29].

## 3. Application of SiMS Microscopy in Cell Biology, Biophysics and Pharmacology

Direct visualization of single-molecule proteins in live cells has revealed unexpected molecular behaviors that are not consistent with prior knowledge from in vitro biochemical studies. For examples, at steady state in vitro, actin assembles at the barbed end (plus end) and depolymerizes at the pointed end (minus end), which is referred to as “treadmilling”. Lamellipodia, the sheet-like structure which cells extend during migration, contain a highly dense actin meshwork [30]. In lamellipodia, actin filaments are mostly oriented with their barbed ends facing the cell edge [31], and the actin meshwork is constantly conveyed to the cell center by the retrograde actin flow [10,32]. These observations led researchers to hypothesize that actin undergoes treadmilling in a manner that actin polymerizes at the leading edge and depolymerizes at the base of lamellipodia [12]. On the other hand, observations of single-molecule actin speckles revealed that filament turnover in the lamellipodial actin network is very rapid, as nearly one-third of filaments have short lifetimes of less than 10 s, and actin turnover distributes broadly throughout lamellipodia [19,33]. As discussed in our recent publication [34], these observations suggest that treadmiling does not solely account for the actin filament turnover in vivo.

Capping protein (CP) regulates actin polymerization by binding the barbed end of an actin filament, which blocks assembly and disassembly of actin subunits [35]. In vitro, CP tightly binds to the barbed end with a *Kd* of 0.06–1 nM [36,37,38]. Surprisingly in lamellipodia, SiMS analyses revealed that the dissociation of EGFP-tagged CP (EGFP-CP) occurs at 0.58 s^−1^, which is 100-times faster than the dissociation of EGFP-CP from the barbed end at 0.005 s^−1^ in vitro [3]. Attenuation of the fast dissociation of CP upon blockade of the actin depolymerizing activity suggests that frequent filament severing might take place in the lamellipodial actin networks. Evidence from SiMS analysis on the cofilin cofactor AIP1 [27] also supports the existence of a non-treadmilling actin turnover mechanism by which a substantial amount of actin might disassemble near the barbed end of the filament [34].

SiMS data have been used in quantitative mathematical models to investigate how complex actin dynamics is coordinated in vivo. Ryan et al. proposed a model postulating that a diffusive, autocatalytic activator promotes actin polymerization; filamentous actin accumulation in turn inhibits further activator accumulation [39]. Simulations of the model reproduced the pattern of actin polymerization seen in SiMS experiments in the cycle of lamellipodial protrusion and retraction. The Arp2/3 complex, which is the key nucleator of branched actin meshwork in lamellipodia [30], accumulates several seconds before actin filament accumulation [39]. This observation suggests that the Arp2/3 complex may participate in an activation mechanism. 

SiMS analysis is often capable of revealing the rapid response of molecules to pharmacological treatment. Vemurafenib is a BRAF (v-raf murine sarcoma viral oncogene homolog B1) enzyme inhibitor for the treatment of patients who have metastatic melanoma. The compound was developed to inhibit BRAF^V600E^, a cancer-associated mutant of BRAF. However, vemurafenib can paradoxically activate the Ras/RAF/MAPK pathway via activation of CRAF (v-raf-1 murine leukemia viral oncogene homolog 1) through the formation of dimeric RAF complexes [40,41]. Such unexpected allosteric effects of target-based drugs have attracted substantial attention, because these allosteric effects may change the therapeutic effects of the drugs or cause unexpected side effects. Prior to the findings with vemurafenib, our study employing SiMS imaging approach reported that imatinib, a target-based drug against chronic myelogenous leukemia, induces allosteric effects on its target molecule, Abelson kinase (c-abl) [42]. SiMS imaging revealed that rapid cell-edge translocation of c-abl was induced by imatinib. We compared imatinib-induced molecular behaviors of c-abl mutant proteins, which provided clues to solving the conformational changes of c-abl upon imatinib binding [42]. As shown in this example, direct viewing of molecular behavior would facilitate solving the action of target-based drugs unambiguously.

## 4. Semi-Automatic Imaging Analysis Tool, Speckle TrackerJ

**Figure 2:**
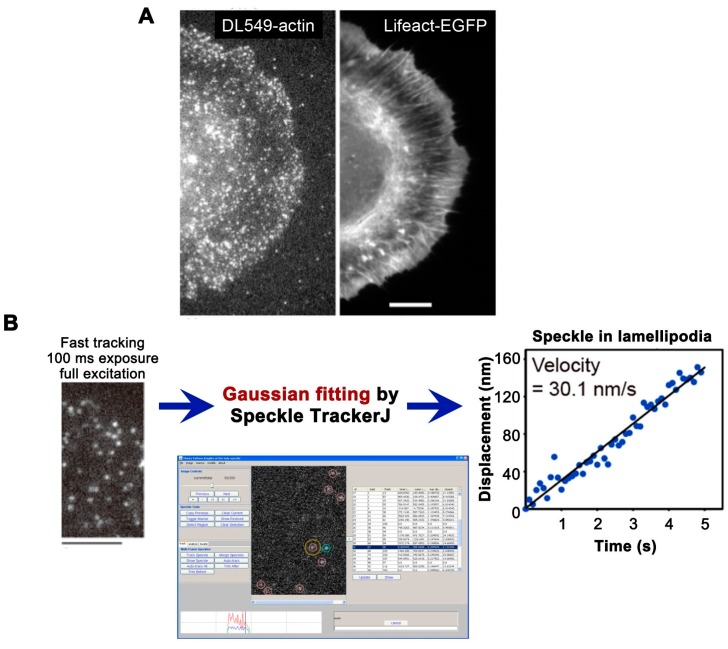
(**A**) Live Electroporation-Based SiMS Microscopy (eSiMS) image of DL550-actin in lamellipodia of XTC cells loaded with DL550-actin by electroporation (left) and actin structures visualized by Lifeact-EGFP (enhanced green fluorescent protein) (right). Bar = 10 μm; (**B**) Nanometer-scale displacement analysis in cells with DL549-actin. DL549-actin speckles are acquired with a 100 ms exposure time and a full 100 W mercury excitation (left), and then localization of the SiMS centroid is determined by using the Gaussian fit model of Speckle TrackerJ (middle). The graph (right) is displacement plot of the central position of a DL549-actin SiMS in lamellipodia in the series of images acquired as the right image. Bar = 5 μm. Modified from Yamashiro et al., 2014 [33].

To assist in the analysis of SiMS data, the imaging analysis tool, Speckle TrackerJ [43] has been developed by our collaborator Dimitrios Vavylonis and his colleagues at Lehigh University. The tool is a freely available open source ImageJ plugin [44]. Speckle TrackerJ enables the recording of the position, appearance and disappearance of numerous speckles through time. The software is designed to allow manual operation of a number of error-correction commands in combination with computer-based particle tracking. With the user’s supervision/assistance, Speckle TrackerJ greatly helps the collection of reliable results from images of mixed qualities. In addition, measurement of speckles’ centroids by using the two-dimensional Gaussian fit model of Speckle TackerJ facilitates high-resolution tracking of nanometer-scale displacement analysis [33] (Figure 2B). The method to use Speckle TrackerJ is described on the above website, and data analyses using Speckle TrackerJ were introduced in our recent publications [22,33,43,45]. Other automatic particle detection and tracking software currently available from various resources can track individual molecules fairly precisely in ideal situations, but the frequency of tracking errors often becomes intolerable, influenced by the variations in clarity and density of single-molecule images.

## 5. Easy, Efficient and Electroporation-Based SiMS Microscopy (eSiMS)

In 2014, we introduced a new SiMS method, which is easy-to-use for researchers without prior background in SiMS and that achieves high spatiotemporal resolution [33]. We call the new method eSiMS. In comparison with eSiMS, the original SiMS method demands experience to find cells containing EGFP-tagged probes at the optimal level [19]. Although the low expression levels of genetically-labeled proteins are achieved under the control of the defective CMV promoter, only a minor population of cells expresses EGFP-tagged proteins at a sufficiently low level. This technical difficulty for SiMS has prevented wider use of this technique in the research community.

In eSiMS, we employed an actin probe that is chemically labeled with a red fluorescent DyLight 550 dye (Thermo Fisher Scientific, Waltham, MA, USA) on lysine side chains. Electroporation-mediated delivery of DyLight 550-actin (DL550-actin) enables the labeling of cells with 100% efficiency at the optimal low density (Figure 2A). Note that excessively high density causes overlapping of SiMS, which prevents accurate tracking of individual molecules. Too low a density makes SiMS imaging difficult due to the photobleaching of fluorophore during the finding of cells process and focus adjustment. In addition, too low a density limits the number of observations of speckles in each experiment. An example of optimal density for SiMS analysis are shown in Figure 2A,B, whereas the “optimal density” could be variable depending on the purpose of SiMS measurement. Furthermore, DL550-actin labels cellular actin networks with improved photostability and brightness compared to EGFP-actin. These favorable properties of DL550-actin the extend time window of the eSiMS analysis. In combination with the Speckle TrackerJ software, the eSiMS method enables in vivo nanometer-scale displacement analysis with a low localization error of 8–8.5 nm at 100 ms exposure time (Figure 2B). The basic protocol of eSiMS microscopy is introduced in our recent publications [22,33]. Comparison between the original SiMS method and the eSiMS method is summarized in Table 1. Note that DyLight 550 is the replacement of discontinued DyLight 549 that we had used in our previous study [33].

The actin network at the cell periphery continuously moves toward the central region, namely the retrograde actin flow [10,32]. The retrograde flow is believed to promote cell edge protrusion when linked to focal adhesions. Before the development of eSiMS method, detailed analysis on the retrograde flow was missing due to rapid actin turnover, the use of GFP-actin, the lack of appropriate analysis algorithms, and technical difficulties. Direct viewing of individual actin filaments by eSiMS microscopy reveals the actin flow velocities including formin-based, short-lived and locally modified actin populations [33]. By using eSiMS, we readdressed the remaining question in the previous quantitative fluorescent speckle microscopy (qFSM) study [46] which has caused intensive debate over the fidelity of qFSM [47,48]. Our eSiMS analysis [33] failed to reproduce the previously proposed coexistence of actin populations with different speeds in lamellipodia [46]. In addition, we revealed how focal adhesions influence local retrograde flow in lamellipodia in detail by using eSiMS [33,49].

Usage of chemically labeled probes in the eSiMS method extends availability of fluorophores to a wide-range of excitation/emission wavelengths. Near infrared (NIR) emissive dyes have advantages in the imaging of thick cells and tissues, because fluorescence imaging in the long-wavelength region facilitates low autofluorescence background, low phototoxicity to cells, and deep tissue penetration [50,51]. In the accompanying article, we introduce a new NIR actin probe with excellent photostability which is comparable to that of DyLight 549.

The new eSiMS microscopy introduced here makes it easy to use live-cell single-molecule imaging with a very high spatiotemporal resolution. With the methodological advantage, the eSiMS microscopy will help researchers extend the application of single-molecule analysis. For instance, the principles of the eSiMS method are applicable to many other proteins for monitoring various molecular dynamics in live cells. In addition, a new NIR actin probe introduced in the accompanying article will extend single-molecule analysis to monitoring actin remodeling processes in deep cellular structures with three-color imaging. The eSiMS microscopy with NIR probes will be applicable for monitoring single-molecules three-dimensionally. To carry out the three-dimensional SiMS analysis, tracking and analyzing molecular behavior, while reducing photodamage during SiMS imaging in multiple focal planes, will be some of the new challenges.

## Figures and Tables

**Table 1:** Comparison between the original SiMS method [19] and the eSiMS method [33].

	Original SiMS	eSiMS Method
**Actin probe**	EGFP-actin	Organic fluorescent dye-labeled G-actin
**Delivery to cells**	Transfection	Electroporation
**Ease of finding cells for SiMS imaging**	Slightly difficult: It demands experience to find cells expressing EGFP-actin at an optimal level.	Easy: Electroporation enables the incorporation of probes into almost 100% of cells at an optimal level.
